# The Undiagnosed Diseases Program Integrated Collaboration System (UDPICS): One Program’s Experience Developing Custom Software to Support Research for Complex-Disease Families

**DOI:** 10.3390/children2030342

**Published:** 2015-07-31

**Authors:** Jessica Guzman, Elizabeth Lee, David Draper, Zaheer Valivullah, Guoyun Yu, Murat Sincan, William A. Gahl, David R. Adams

**Affiliations:** 1 Undiagnosed Diseases Program, National Institutes of Health, Bethesda, MD 20892, USA; E-Mails: jessica.guzman@nih.gov (J.G.); elizabeth.lee3@nih.gov (E.L.); draperd@mail.nih.gov (D.D.); zaheer.valivullah@nih.gov (Z.V.); guoyun.yu@nih.gov (G.Y.); 2 National Institute of Dental and Craniofacial Research, Bethesda, MD 20892, USA; E-Mail: sincanm@mail.nih.gov; 3 National Human Genome Research Institute, National Institutes of Health, Bethesda, MD 20892, USA; E-Mail: gahlw@helix.nih.gov

**Keywords:** informatics, UDPICS, Undiagnosed Diseases Program, Undiagnosed Diseases Network, rare disease, collaboration software

## Abstract

The Undiagnosed Diseases Program (UDP) was started in 2008 with the goals of making diagnoses and facilitating related translational research. The individuals and families seen by the UDP are often unique and medically complex. Approximately 40% of UDP cases are pediatric. The Undiagnosed Diseases Program Integrated Collaboration System (UDPICS) was designed to create a collaborative workspace for researchers, clinicians and families. We describe our progress in developing the system to date, focusing on design rationale, challenges and issues that are likely to be common in the development of similar systems in the future.

## 1. Introduction

The NIH Undiagnosed Diseases Program (UDP) was started in 2008 with two goals: finding diagnoses for patients who remained undiagnosed despite extensive medical workup, and conducting research based on undiagnosed disease [1]. To date, the program has received approximately 10,000 inquires, reviewed more than 3400 charts, and accepted more than 800 patient/families. Approximately 40% of cases are pediatric. Diagnoses have been offered in approximately 25% of cases. The remaining 75% of cases are closely examined for clues that might shed light on disease etiology and/or mechanism. Potentially disease-related characteristics are sought using in-depth phenotyping, genome-scale sequencing (exome or genome), metabolomics, glycomic screening and other methods. Such characteristics are used as starting points in pursuing translational research. In many cases, the program attempts to identify subject-expert collaborators to pursue research leads.

The UDP cohort has some general characteristics. They are the result of both the types of families who contact the program and the program’s subject selection practices. For instance, over 50% of accepted applicants have neurological symptoms as their primary disease manifestation. Most applicants present with extensive medical records, documenting years of clinical testing, consultation, and supportive care. Despite these commonalities, there are few subjects who can be grouped together based on similar clinical presentations. The wide spectrum of presentations presents two particular challenges for tracking patients through the program. Firstly, each participant family follows an individualized path from initial application through to research establishment. Secondly, research efforts for many participants cannot make use of the associative power of large cohorts and may take years to complete. In consequence, the rate of enrollment exceeds the rate of case completion (successful definitive diagnosis). A large number of individual research projects have accumulated over the life of the program. Tracking and managing UDP-associated projects is a major undertaking of the program.

The set of software tools we have developed to manage these data is called the Undiagnosed Diseases Program Integrated Collaboration System (UDPICS). The system is in a constant state of development and would require substantial configuration to be implemented at a new site. However, all of the components are available to interested persons. The broader aim of this paper is to describe the process of creating a collaborative workspace around individuals and families with rare and complex illnesses. We hope that this description will stimulate discussion about the role of informatics in bridging the gap between patients, clinicians and researchers.

### 1.1. Stakeholders in the Care of Complex Undiagnosed Patients

**Figure 1:**
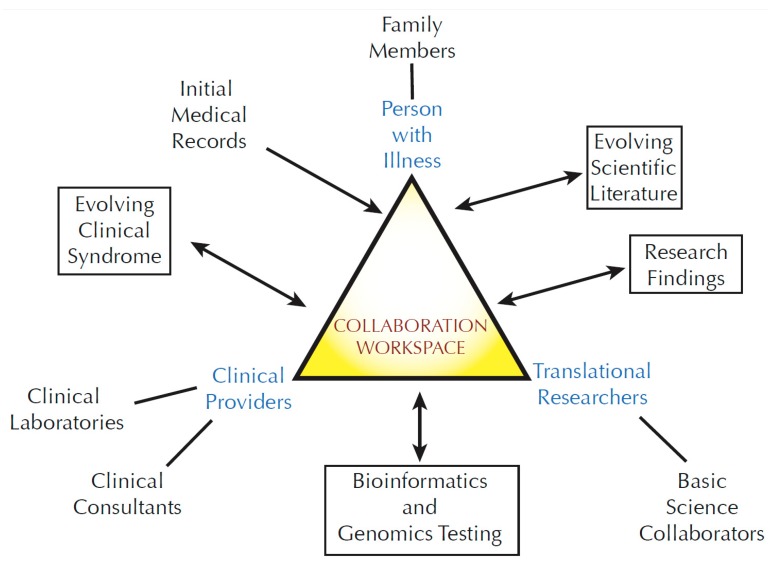
The community of stakeholders associated with translational research.

A person with an undiagnosed disease may become associated with a large number of stakeholders: primary medical providers, subspecialty providers, medical consultants, clinical research collaborators, basic science collaborators, and clinical testing sites (Figure 1). Genetic studies further expand this group by adding additional family members and bioinformatics professionals. The affected person’s story evolves over time, marked by the accumulation of a large and varied body of data. Communication of these data among the involved stakeholders becomes progressively challenging. In an optimal situation, a primary care provider or complex disease specialist might act as a coordinator. However, the addition of basic or translational research adds a new layer of complexity. Traditionally, a single physician-scientist carries out coordination of research efforts. Such research programs generally occur when there is a fortuitous matching of a patient phenotype with the interests of an established research group. That model does not scale well in an era where genome-scale sequencing and other emerging techniques are creating a large number of potentially new rare disease candidates in need of research follow up. For the UDP, recordkeeping around individual participants overwhelmed our resources within a year or two of starting the program.

### 1.2. UDPICS Requirements *versus* Existing Software Packages

Clinical informatics is an established field and multiple software packages are available. New software is introduced on a regular basis. A strong argument against creating any new system is overlap with existing functionality. In addition, new systems have the potential to create new standards and procedures that may impair communication rather than facilitating it. From our perspective, existing clinical information systems fell into several categories:

1. Electronic Medical Record (EMR) systems, e.g., Cerner (Cerner Corporation, Kansas City, MO, USA) and Epic (Epic Systems Corporation, Verona, WI, USA)
2. Systems optimized for clinical trials, e.g., REDCap [2]
3. Laboratory Information Management Systems (LIMS)
4. Data warehouse and data harmonization initiatives, e.g., i2b2 [3]

Within each of these categories, there are examples of exemplary functionality, usability and power. However, a number of specific UDP requirements were not addressed by any combination of existing software packages. We required a solution that would provide data access to multiple, disparate stakeholders, with permission and access defined separately for each project. Data entry and storage needed to accommodate a balance between structured database entries and bulk digital data ranging from proprietary EEG output to scanned images. The system needed individualized workflows, reflecting the observed diversity of paths through our system.

#### 1.2.1. Rapid Flexibility and Existing Electronic Medical Records Systems

Application Programing Interfaces (APIs) allow direct digital connections between computer programs. While APIs are in no way unique to UDPICS, existing systems vary in the speed and expertise requirements of deployment. We required that UDP staff be able to set up connections with outside data sources without requiring extensive (and often expensive) help from software developers. Hospital managed EMRs frequently limit user configuration and use of API subsystems in an effort to protect data integrity and/or as part of a profit model. UDPICS provides a standard REST API and is able to make API-based queries to other systems. The architecture for these connections is integrated with stored data elements in a way that allows for customizable data exchange without the need for extensive software development resources. All such customization is available to our local staff and does not require intervention from the software vendor.

#### 1.2.2. Heterogeneous Workflows and Clinical Trials Software

The term “workflow” is commonly used to describe a tool for documenting a process. We use the term workflow to describe a connected series of states. A state is any temporal characteristic of a process being tracked. A simplified example might be a patient status workflow with three states:

1. Subject has applied.
2. Subject has been accepted.
3. Subject has been seen.

**Figure 2:**
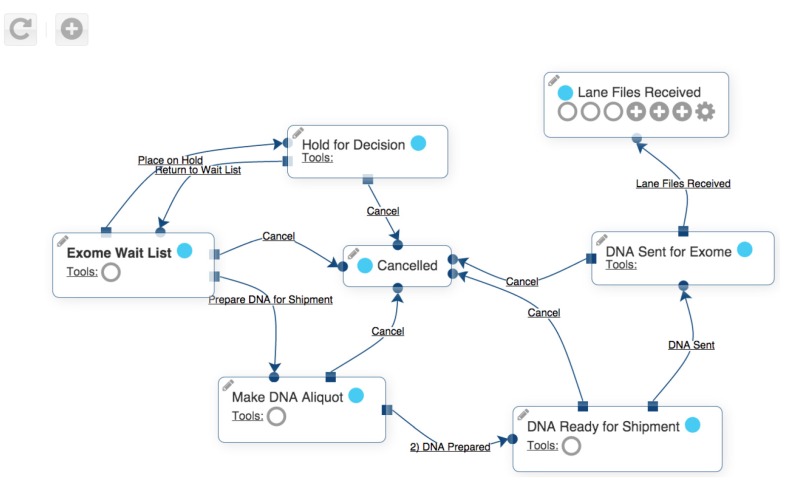
Example of Undiagnosed Disease Program Integrated Collaboration System workflow.

**Figure 3:**
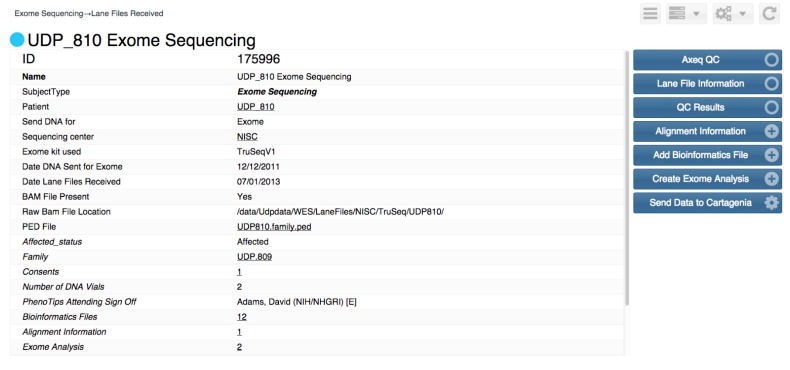
User view of an example of Undiagnosed Disease Program Integrated Collaboration System workflow.

One or more instances of such a workflow can be assigned to each incoming patient. Workflow states can be complex with many states and transitions. Figure 2 shows a workflow for sending exome sequencing specimens. Each box is a state, and each arrow is a transition from one state to a different state. Figure 3 shows the user interface used to navigate between states. This type of workflow is available in many of clinical and research support programs.

Workflows in the UDP required a number of specific features. Firstly, workflows needed to tolerate extensive versioning. The versioning requirement arose from the fact that UDP processes evolve rapidly over time. Such evolution contrasts with the fixed protocols that are typically required for clinical trials. Secondly, individual subjects often required multiple copies of workflows. An example would be the exome workflow, where multiple generations of technology might be applied to a single subject. Thirdly, the complexity of some workflows required special consideration. Custom scripting was frequently needed to work with data in a state-associated context, and to perform procedures associated with transitions between states. Finally, the frequent generation and modification of workflows became cumbersome to perform using a menu-driven approach.

The clinical research software REDCap is an example of existing software for clinical trials. It has extensive features and a large user base, being deployed for thousands of projects worldwide. In fact, some authors of this paper are implementing REDCap for a number of their own therapeutic trials. REDCap was not a good solution, however, for the UDP. REDCap workflows are best suited for infrequent modification for a given study. While it is possible to create new workflows that link to previously entered elements, creating such linkages is cumbersome to perform on a regular basis. UDPICS incorporates a graphical user interface and modular elements that can be rapidly and arbitrarily grouped together into new workflows. In particular, *ad hoc* workflows for individual collaborations can be assembled on the fly as new research initiatives are established.

#### 1.2.3. Diverse Functionality and Laboratory Information Management Systems (LIMS)

LIMS software is designed to record data from a research laboratory. Components may include laboratory notebook, bio-repository, colony management, productivity and other functionality. Many of these capabilities were required by the UDP. In fact, UDPICS currently uses some external-but-integrated components for discrete tasks such as sample storage and tracking. The commercial software that we started with (“LIMS 24/7”) was essentially a flexible LIMS platform. A major consideration for choosing this software was willingness by the commercial developer, RURO Inc., to modify their product. Many features, including a collaborator server system, a set of integrated of external software programs, pedigree functionality, phenotyping entry and analysis software, and a graphical interface for workflow construction were added during the course of development.

#### 1.2.4. Practical Aspect of the Current State of Medical Records and Data Harmonization Initiatives

The next challenge faced by the UDP involved documentation. Most UDP patients provide their prior medical records in the form of printed materials and photocopies. Even electronic records often arrive as scans, folders of loose image files and video files from cell phones. The formal medical records come from a wide variety of providers and institutions, essentially preventing the establishment of effective electronic data transfer given practical time and cost constraints. Our informatics system, therefore, needed to capture records using a variety of methods. For scanned records, raster images are stored as is with minimal added metadata. Digitally stored records in unfamiliar formats are treated similarly. Some records with standardized or otherwise well-documented formats can be imported into structured database tables. Data warehousing and harmonization initiatives such as i2b2 will hopefully chart the way to a future where data can move more easily between clinical and research environments. In the current medical records environment, however, the reality is a mixture of file types and formats. For the UDP, efforts to transform incoming records into searchable, well-formatted forms needed to be balanced against associated costs.

### 1.3. Commercial *versus* Open-Source Software Package

The benefits and liabilities of open source *versus* commercial software have been debated widely. We elected to use commercial software for several reasons (Of note, none of the authors has a financial interest in any of the companies described in this paper). Firstly, the company we chose, RURO (RURO, Inc., Frederick, MD, USA), was willing to make extensive changes to their product. Secondly, working collaboratively, the software was customized for UDP at a price much lower than would be charged for developing an entire system. Thirdly, the cost of developing UDPICS with a commercial partner was less expensive (on the order of $400,000) than the alternative of hiring software developers to modify existing open source software. Fourth, the priority of the UDP being translational research and clinical work, we were not able to commit to ongoing support of our final software for users outside the UDP. Working with a company allowed us to concentrate on optimizing and using the software rather than on maintenance and future support.

A complex community develops around families and individuals with complex disease. The sphere of participants increases when the family becomes involved in translational research. For the UDP, this collaboration generally occurs for a single family (rather than a cohort of families with similar illnesses). Communication with the stakeholders is an ongoing process because clinical and research information evolves over time. The three principal participants (the person with the illness, the translational researchers and the clinical team providing medical support) require a mechanism to conduct and document ongoing collaboration. Optimally, such a collaboration workspace will have an asynchronous component—data can be entered and read at arbitrary times rather than being limited to pre-arranged conferences.

The UDPICS exome sequencing workflow has seven states indicated by boxes. Arrows between the boxes indicate transitions between the states. Symbols within the boxes indicate various tools that are available to the user while the subject is in a particular state. States may be duplicated. For instance, exome sequencing might be repeated if newer technology becomes available.

From the user’s perspective, each workflow state appears as a web-browser page with information in the left panel and optional activity buttons on the right. Some buttons transition the workflow to different states, while other buttons allow data entry, data lookup and other functionality.

## 2. Results and Discussion

### 2.1. Infrastructure and Initial Setup

Much of the basic framework of UDPICS is derived from requirements of the RURO product (Limfinity) on which UDPICS is built. The physical hardware required by the program is a modest server on the order of a high-end desktop computer. We set up our server in a Linux environment hosted on a virtual server. The application framework consists of a Ruby on Rails application with a PostgreSQL database backend. Other configurations (and backend databases) are possible and will likely be required as the system grows in size. Users interact with the system using a web-browser. Printers and other physical devises can be connected to the system using standard network protocols. A pervasive challenge of using any system with Personally Identifiable Information (PII) is security and privacy. Our environment requires two-factor login (a card plus a pin number) for systems with clinical data. However, setting up such login mechanisms for all current and future collaborators was not practical. We addressed this problem by setting up a mirrored collaborator server of the software outside our institutional firewall. Only non-PII data is transferred to the collaborator server allowing for single-factor (user/password) login by external collaborators.

### 2.2. External Systems Connected to UDPICS

UDPICS connects to a variety of external systems. For the sake of convenience, we purchased some of these from RURO as additional products. Examples include a biobanking program (“FreezerPro”) and a model organism tracking program (“EZColony”). The remaining systems comprise a mixture of products from other vendors plus internal UDP software. We have developed a customized exome alignment and annotation pipeline, which is hosted by the data services and genomics company Appistry (Appistry, Inc., St Louis, MO, USA). Progress of samples through the pipeline is tracked by UDPICS. Our exome analysis pipeline utilizes a combination of custom in-house scripts and a commercial system from Cartagenia (Cartagenia Inc., Cambridge, MA, USA). The Cartagenia component allows for expanded visualization, recording and access to analytic procedures and data. Progress through the analytic pipeline, including file transfers, analytic status and final data recording is handled by UDPICS. In this manner, individual users can login to one system to view and work with data collected from many sources. At present, we do not have a direct connection with the EMR of the NIH Clinical Center where our study participants are clinically evaluated. We have instead prioritized phenotyping—a process of defining a subject’s medical condition in a standardized ontology. To accomplish phenotyping, UDPICS is integrated with the software PhenoTips [4]. PhenoTips opens within UDPICS and provides an interface for phenotype coding using the Human Phenotye Ontology (HPO) [5]. HPO is a hierarchical phenotype ontology that includes mappings to both other human ontologies and to model organism phenotype ontologies. As an example of the utility of the HPO encodings, UDPICS integrates the program PhenoGrid. PhenoGrid allows for comparison of the phenotypes of individuals with in the UDP and for finding model-organism genes associated with mapped phenotypes [6].

### 2.3. Connection with Laboratory and Other Personnel within the NIH

In addition to electronic connectivity, the UDP requires interaction with a substantial network of clinical, bioinformatics, laboratory, legal, and clinical support people both within the UDP program and across the Clinical Center campus. For example, all shared bio-specimens require using a formal Material Transfer Agreement (MTA). MTA establishment involves close communication with the technology transfer and legal offices of the NIH intramural program. These communications have been set up as UDPICS workflows. Requests for MTAs by clinicians and researchers are automatically sent to the appropriate technology transfer staff along with the information that is required to execute the MTA. In practice, multiple MTAs may be established over a week’s time. Once the MTA process has been signed off by all relevant parties (also tracked by UDPICS), the samples associated with the MTA are flagged to allow further work on a given family to be considered in the context of documented collaborations. The stored data is also available for audits of freezer samples and other institutional quality assurance initiatives. Other examples include tracking of clinical grade preparation of DNA specimens, requests for research laboratory work (Sanger validation of DNA findings, preparation of plasmids for collaborators, DNA splicing studies for potential splice variants, *etc.*).

### 2.4. Connections with External Laboratories

Communications with external laboratories have been set up primarily through the UDPICS collaborator server. For instance, metabolomic and glycomic screening has been performed on urine, blood and cerebral spinal fluid for a cohort of approximately 200 study participants. Collaborators performing the screening assays upload returning data into the UDPICS collaborator server. From there, it can be synced to the main UDPICS instance for use by program clinicians and researchers. Staff members associated with a given case are notified by email on data upload, so that they can examine the data and participate in the interpretation process.

### 2.5. Connection with File Systems for Large File Storage

Genomic data involves files that are large relative to current network transfer speeds. In particular, short read data in the BAM file format are on the order of 10 Gb for exomes and 100 Gb for genomes. This data is not stored directly in the database, but resides on a separate file storage system. Standardized file pathnames are used to record the location of files.

### 2.6. Current Use by the UDP

UDPICS currently has 101 users within our program. We have entered approximately 1.5 million data values. System infrastructure contains 91 workflows and 2209 data field types. The software is in routine use for a range of UDP activities, including (but not limited to):

– Program application tracking, including chart review for prospective applicants
– Genomics tracking including exome sequencing, sample tracking, alignment, analysis, interpretation, variant prioritization and data sharing for external collaborators
– MTA tracking
– Electronic laboratory notebook functionality for documentation of basic-science data and projects
– Patient-specific communications between UDP staff/collaborators, including email integration
– Sample processing and tracking, soon to include medical grade Clinical Laboratory Improvement Amendments (CLIA) samples
– Access by external collaborators to patient phenotypes, genotypes in a subject and project specific manner
– Phenotype collection and curation
– Submission of data to public databases such as dbGaP and PhenomeCentral

**Figure 4:**
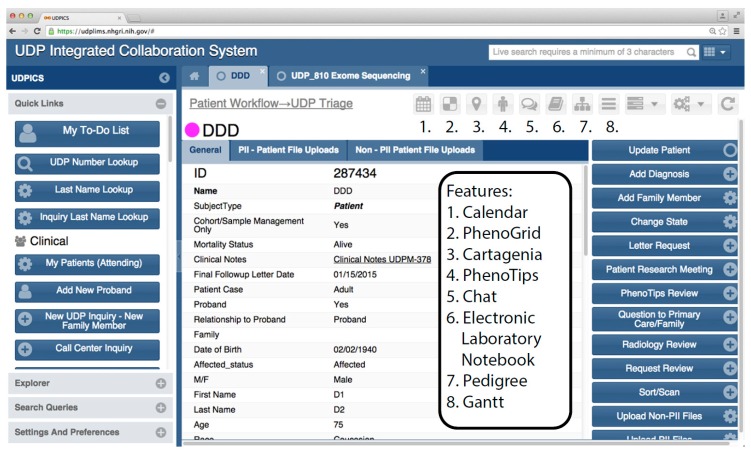
Typical UDPICS user interface webpage.

Figure 4 shows a typical user interface screen with some of its components.

### 2.7. Cost

The true cost for a system like UDPICS is somewhat difficult to calculate because different institutions cover infrastructure costs differently. In addition, cost for development of a system is different from the cost of maintenance. Table 1 lists some approximate costs. These are based on our particular study cohort, institution and other internal resources.

**Table 1:** Approximate costs associated with UDPICS for the UDP.

Item	Approximate Cost	Notes
System Development	$400,000	One time, initial expenses
Software Licensing ^a^	$100,000	Yearly
Support from Vendor	$50,000	Yearly
Infrastructure ^b^	$150,000	Yearly
Initial Security Assessment	$20,000	One time expense
Security Monitoring	$2,000	Yearly
Local Support Personnel ^c^	$250,000	Yearly

^a^ Includes all RURO products that we are using (Limfinity, FreezerPro, EZColony, Sciency), but not products from other connected systems such as genomic analysis software from Cartagenia Inc.; ^b^ The infrastructure cost estimate is based on a combination of factors including data storage, expert maintenance and hosting of servers. At one time, the UDP planned to host UDPICS at a commercial vendor. The cost associated with that hosting, which included security and system-migration expenses, was approximately $300,000 for three years; ^c^ Support estimate includes primarily configuration (internal work) as opposed to maintenance of infrastructure. Included are data curation; ongoing maintenance and generation of workflows; connections with external systems; and, bulk, manual data entry/manipulation. The estimate does not include routine data entry by UDP staff.

Users of UDPICS have access to extensive functionality. New features are added frequently as the system is refined and as new research initiatives are implemented. Buttons (square boxes) on the left panel are available at all times and can be shown or hidden depending on the specific role(s) of the user. Buttons on the right side are context dependent and change with the specific work being done at any time. Smaller buttons (numbered) are used to launch special features and external software. Some software, for instance PhenoTips, opens as a window within the UDPICS application. Other software, for instance Cartagenia, opens in an external browser window.

### 2.8. Outcome

The overall efficacy of UDPICS is difficult to quantitate. It did not replace a similar system to which it can be directly compared. The system began and continues to experience rapid ongoing development as a result of a currently marked evolution in the medical profession’s approach to undiagnosed disease. Genome scale sequencing, for instance, has moved from an expensive, large-scale research methodology to a clinical commodity. Communication between rare-disease researchers is evolving rapidly as exemplified by participants in the Matchmaker Exchange [7]. Nonetheless, the following vignettes describe cases where the functionality of UDPICS has been successfully deployed to the benefit of study participants.

#### 2.8.1. Patient Application Process

During the early years of the UDP we were overwhelmed with the handling and tracking of patient admissions. Families would contact us by phone, email or simply by sending large collections of variably sorted medical records. Each application was assessed to determine whether sufficient data was available to review the case for potential study enrollment. Initial contact was followed up by an individually drafted letter listing any missing medical information. Construction of a complete medical record often included numerous communications with the family and their care providers over 4 to 12 months. Once assembled, paper copies of the medical record were distributed to clinical specialists and other consultants for review. After the reviews were complete, reviewers would send back comments and suggestions to be added to the applicant’s medical file. Paper copies of the medical records created for the reviewers would have to be stored or securely destroyed. For accepted applicants, additional copies of records would be delivered to medical consultants before the enrolled subject was admitted to the NIH Clinical Center. New information arriving from the family between the time of acceptance and admission would have to be copied and distributed in a similar manner.

UDPICS has allowed us to move to a different model where patient status is tracked from the time of application forward through admission to various stages of post-admission follow up. Events that used to be recorded in paper charts are now available online for searching and/or remote access. Scanned records are placed in the UDPICS system, reducing the need for paper reproduction of protected health information. Frequently utilized consultants are given direct access to UDPICS so that their review process can be conducted without the need for digital file transfer. Communications with the family are recorded in the system, a particularly useful resource for families that are contacted infrequently during the record accumulation process. Major stakeholders in the review, clinical evaluation and follow up process are recorded so that questions about a given family can be directed to the appropriate person. Benefits of the system have included reduction of lost records, reduced time collecting the data needed for patient inquiries and an improved ability to generate overall statistics for the review process.

#### 2.8.2. Exome Sequencing Results

Exome sequencing grew from obscurity to routine practice over the first seven years of the UDP’s existence. Over this time, results from exome sequencing have accumulated along with associated data. Genome-scale sequencing results for UDP patients are often research leads rather than clear diagnoses. DNA variants are associated with extensive metadata including the circumstances and methods of analysis, the program’s level of interest/confidence in any given finding and the most recent information about current collaborations associated with the case. In the early days of the UDP, conversations about variants occurred on an *ad hoc* basis. If a collaborator wanted information about the analysis method used to find a variant, they would have to identify the associated analyst and discuss the case via email or phone. Separate conversations were needed for information about the clinical presentation and biosamples availability. Furthermore, there was no specific place to record conversations about cases and no designated place to store results produced by a collaborator. As the number of cases grew, the recordkeeping associated with these activities became essentially impossible to keep up with.

UDPICS has successfully addressed these problems. A recent research-funding announcement (RFA) for DNA variant functional studies illustrates our current process. Individual sequence variants are added to a prioritized variant list. Each variant links to a specific analysis, analyst(s) and rich set of data about the methods and annotations used to find the variant. A subset of these variants was designated for inclusion on the list of variants to be included in the RFA. Individuals wishing to respond to the RFA were given access to the UDPICS collaborator server. After logging in to the collaborator server, RFA respondents had access to a wide range of de-identified information about the case. Examples included a pedigree, a phenotype encoded in HPO terms and de-identified clinical narratives, a set of variant metadata, and a list of available biosamples. Of note, each of these types of data was entered into UDPICS by a different group of UDPICS users over the course of the subject’s interaction with the UDP. The result has been a qualitative improvement in the types of questions posed to the UDP staff by RFA respondents. Questions for prior RFA announcements centered around requests for basic information, whereas questions for the most recent RFA announcement were generally nuanced questions about clinical presentation or other features of particular cases.

## 3. Discussion and Conclusions

UDPICS is currently in active use by the UDP. Portions of the program, as highlighted by this paper, have been implemented to some degree. We consider UPDICS to be a research project rather than a completed package: it has been under active development since standing up the first production version. Components of UDPICS can be purchased from our various commercial collaborators. However, UDPICS as a complete system comprises a large set of configurations unique to our own physical infrastructure, staff and other resources. Analogous to a hospital installing a new EMR, producing the full capabilities of UDPICS in a new site would require a substantial investment in setup.

UDPICS was developed in response to several key features of the UDP. Our study population consists of individual families with complex medical problems. We attempt to transition unsolved cases into translation research, creating a need for a collaborative workspace for ongoing communication between clinicians, researchers and families. The heterogeneous nature of our study cohort has resulted in an individualized process for any given family. Our requirements for UDPICS may have substantial overlap with patient care/research interfaces in many settings. Examples include tertiary care centers and diagnostic referral centers tied to research centers. UDPICS itself may be a solution for some settings. We currently have a demonstration server that is available for interested centers to test (contact corresponding author for relevant information). For other sites, the development of new or differently customized software may be a consideration. In either case, relevant considerations will likely be cost, establishing development priorities, privacy and the timing of development *versus* deployment.

### 3.1. Cost

Planning for the development of a system like UDPICS requires assessing costs beyond basic infrastructure and licensing. For us, storage, security and staffing costs were larger than licensing costs. This fact was an additional part of our deliberations with regard to open source software. Open source software may be freely available, or available at reduced cost. However, developing new open source tools, and/or making substantial changes to existing ones, may require expensive expertise and infrastructure. If the institution developing the software plans to disseminate it to other centers, there is an additional time and personnel cost for supporting future users. Costs associated with development should be considered carefully and realistically. Estimates should include some flexibility for unexpected problems.

### 3.2. Establishing Development Priorities

Our initial requirements suggested a need for either new software or a substantial modification to an existing software package. Several factors influenced our choice of a starting point for software development. Firstly, we did not feel than any existing open source software had functionality close enough to our needs to form an adequate starting point. Secondly, we did not want to expend resources to construct a user interface. Thirdly, we did not want to create the infrastructure to provide support to potential future users. Selection of a commercial vendor included consideration of cost and an assessment of the willingness of the company to modify their software to meet our specific needs.

Planning of software features and capabilities is a critical step in the process. A commercial vendor may answer “yes” to developing capabilities that are more complex than they might appreciate. Review of ongoing software development is susceptible to “specification creep”, where new feature requests and functionality are added intentionally or unintentionally. This can impact project success by pushing back release dates for updated software. Early adoption of a structured development process, such as agile software development, should be considered [8]. In particular, iterative and incremental addition and evaluation of new features will reduce the chance of the entire process failing secondary to an overly ambitious initial scope of work.

As mentioned, we have worked closely with RURO to modify their software to match UDP requirements. We feel fortunate that our relationship with the company has had a strong collaborative character. The collaboration was based on the extent to which various UDPICS features were required for the UDP’s needs balanced with RURO’s ability to develop features for minimal cost to be used in further commercial applications. A number of features required negotiations about cost to RURO *versus* fee-for-service work. This type of negotiation is likely to be typical in any setting where an external developer (open source or commercial) is utilized. The final set of UDPICS features, then, is a product of UDP financial resources, RURO business priorities and the costs associated with implementing individual features. Features that were deferred for future implementation included a study participant portal, and a portal for the study participant’s primary provider.

### 3.3. Security

Security assessments can be particularly time-consuming especially if institutional precedent does not cover all components of the system. Connected system, for example EMRs, may require that electronically connected systems be subject to matching security policies. A risk assessment of our system yielded a result of Federal Information Security Management Act (FISMA) moderate, requiring two-factor authentication for users with access to personally identifiable information (PII). In addition to the initial security evaluation, ongoing monitoring of the system creates staff and infrastructure requirements. For users with no access to PII, one challenge was a negotiation about the level of separation that the PII and non-PII systems required to adequately protect PII. Separate systems provide a more secure system, but add a requirement for database synchronization. Security rules change frequently, contributing to the expense of maintaining a compliant system.

### 3.4. Privacy

The development of UDPICS highlights several types of privacy considerations. Standards for PII are well established. A less defined but also important user base is the research collaborator. Access to specific research results need to be protected for several reasons. Blinded study designs may require sample blinding. Intellectual property access may need to be isolated to a specific group of users to ensure that time and resource investments are followed by appropriate scientific attribution. Such security requires consideration of both authentication (the user is who they say they are) and permissions (the system knows what a user should and should not have access to). Within established organizations (businesses, universities, hospitals, *etc.*), a system such as UDPICS can inherit authentication from the organization for internal users. Authenticating external users can be more challenging. At present, UDPICS uses single-factor authentication for external users. However, there is the possibility that future requirements may force us to use stronger security. Challenges associated with moving beyond simple passwords include cost and user sophistication. Complex login requirements have the potential to form a barrier to use of the system by users such as patients.

### 3.5. Future Directions

UDPICS does not yet meet all of the original goals set out for the project. We have not yet established a means of including home clinical providers and study participants in the collaborative workspace. While this is a future goal for the project, it poses some significant challenges. Inclusion of study participants will require exposing PII through an external interface. This will likely require a revision of our authentication procedures, and will likely require re-consenting families and/or individuals interested in participating. These challenges are countered by increasing evidence that participation of families has an important role to play in rare disease research [9].

Finally, the UDP is in the process of becoming part of a network of clinical, sequencing and other sites—the Undiagnosed Diseases Network or UDN [10]. As the network begins, individual sites will be exploring novel means of accomplishing tasks that UDPICS facilitates for the UDN. This presents an opportunity for further discussion about optimal strategies for supporting research around single families. Our hope is that, in the future, UDPICS will serve as a model for computational infrastructure in rare disease translational research.

## 4. Materials and Methods

Individual software packages discussed in the article are available from the listed companies. Most of the functionality of UDPICS exists as configuration of these tools as described. Access to an online demonstration server is currently available by request to the communicating author. All study subjects were seen under the National Human Genome Research Institute (NHGRI) Institutional Review Board (IRB) approved protocol 76-HG-0238 under the primary investigator William A. Gahl. Investigations were carried out following the rules of the Declaration of Helsinki of 1975, as revised in 2008, plus other relevant standards.
